# The Theory of Planned Behavior and Disaster Preparedness

**DOI:** 10.1371/currents.dis.4da18e0f1479bf6c0a94b29e0dbf4a72

**Published:** 2017-09-06

**Authors:** Mehdi Najafi, Ali Ardalan, Ali Akbarisari, Ahmad Ali Noorbala, Helen Elmi

**Affiliations:** Department of Emergency and Disaster Health, University of Social Welfare & Rehabilitation Sciences, Tehran, Iran; Red Crescent Society, Iran.; Department of Disaster & Emergency Health, National Institute of Health Research, Tehran University of Medical Sciences, Tehran, Iran; Department of Disaster Public Health, School of Public Health, Tehran University of Medical Sciences, Tehran, Iran; Harvard Humanitarian Initiative, Harvard University, Cambridge, MA, USA; Department of Health Management and Economics, School of Public Health, Tehran University of Medical Sciences, Tehran, Iran; Department of Psychiatry, Tehran University of Medical Sciences, Tehran, Iran; Azad University Consultant Psycholgist, Tehran, Iran

## Abstract

**Introduction:**

Disaster preparedness is defined as actions that ensure resources necessary to carry out an effective response are available before a disaster. Disaster preparedness requires a thorough understanding of the factors that influence performance or nonperformance of disaster preparedness behaviors (DPB). The major aim of this research was to further our understanding of DPB based on the theory of planned behavior (TPB).

**Method:**

This was a cross-sectional study of factors determining of DPB in a representative sample of 1233 Tehran inhabitants. Measures derived from the TPB were obtained in the unprepared and prepared people.

**Results:**

Consistent with the theory, intentions to do DPB could the person predicted from attitudes, subjective norms, and perceived behavioral control with respect to DPB; and actually doing DPB was strongly related to intentions and perceptions of control assessed in the prepared people. Theoretical and practical implications of these findings are discussed.

**Conclusion:**

An effective intervention will not only have to encourage people of the desirability of DPB, but also to provide them with the skills and means to do it. The more strongly they can be made to feel that they have control over DPB, the more likely they are to carry out their intentions. That is, heightened perceived control tends to strengthen people’s motivation to do DPB.

**Key words:**

theory of planned behavior; disaster;  preparedness

## Introduction

A disaster is “a serious disruption of the functioning of a community or a society involving widespread human, material, economic or environmental losses and impacts, which exceeds the ability of the affected community or society to cope using its own resources”^1^. Although the categories and causes of disasters may differ, their impacts are common; therefore, a disaster plan should address disaster impacts^2^. Disaster preparedness is defined as actions that ensure resources necessary to carry out an effective response are available before a disaster, or they can be obtained promptly when needed^3^. Disaster preparedness are preparations and adjustments such as storing food and water, preparing a household emergency plan, preparing an emergency kit, and other activities that reduce risk or injury and damage^4^. Actually, disaster preparedness is a health protective behavior, so the behavioral approaches have taken center stage as a means of it. Even though hundreds of thousands of lives were affected without warning by disasters yearly, most people do not concern themselves by preparing until disaster strikes^5^. Therefore, it has become obvious that a more broad-based effort of behavioral change is required. Effective interventions to promote disaster preparedness require a thorough understanding of the factors that influence performance or nonperformance of disaster preparedness behaviors (DPB).

According to many studies conducted on disaster preparedness, several factors affecting preparedness include: critical awareness^2^^,^^4^^,^^6^, risk perception^7^^,^^8^^,^^9^, preparedness perception^10^^,^^11^^,^^12^, self-efficacy^10^^,^^13^^,^^14^^,^^15^^,^^16^, collective efficacy^16^, locus of control^9^^,^^15^^,^^17^, fatalism^9^^,^^14^^,^^17^^,^^18^^,^^19^, anxiety^4^^,^^17^^,^^20^, previous disaster experience^8^^,^^9^^,^^21^^,^^22^, societal norms^23^, sense of community^24^, community participation and empowerment^25^^,^ 26, optimistic and normalization biases^27^^,^^28^, social trust^29^, perceived responsibility^8^^,^^11^, responsibility towards others^6^, coping style^10^^,^^13^^,^^30^^,^^31^ and available resources^25^^,^^32^.

Several theoretical frameworks can be employed in attempts to deal with behaviors that reduce the risk of natural disasters including: Protection Motivation Theory(PMT)^12^^,^^33^, Person Relative to Event Theory (PrE)^11^^,^^34^, Protective Action Decision Model (PADM)^35^^,^^36^, Social-Cognitive Preparation Model^4^ and Theory of Planned Behavior (TPB)^37^^,^^38^.

To date, there has been no study of people using the TPB to explain variability in DPB. The application of a model that explained a significant amount of variance in intentions and behavior would assist in helping develop interventions to disaster risk reduction.

The aim of this study was to examine the theory of planned behavior and investigate its utility in explaining and predicting the factors associated with DPB.

The TPB is a efficacious framework for investigating antecedents of behavior (Figure 1). A central factor in the TPB is the individual’s intention to perform a given behavior. Intentions are assumed to capture the motivational factors that influence a behavior^39^. Intentions are determined by three preceding motivational factors. The first is the attitude toward the behavior and refers to the degree to which the individual has a favorable or an unfavorable evaluation of the behavior in question. The second predictor is a social factor termed subjective norm; it refers to the perceived social pressure to do or not to do the behavior. The third predictor of intention is the degree of perceived behavioral control which refers to the perceived ease or difficulty of performing the behavior. As a general rule, the more favorable the attitude and subjective norm toward a behavior, and the greater the perceived behavioral control, the stronger should be a person’s intention to perform the behavior under consideration. Intention, in turn, is viewed as one direct antecedent of actual behavior. However, the level of success will depend not only on one’s intention, but also on such partly non-motivational factors as availability of requisite opportunities and resources that represent people’s actual control over the behavior^40^.

The relative importance of attitude, subjective norm, and perceived behavioral control in the prediction of intention, and the relative importance of intention and perceived behavioral control in the prediction of behavior are expected to vary across behaviors and populations^39^.

**Theory of planned behavior (Ajzen, 1991):**
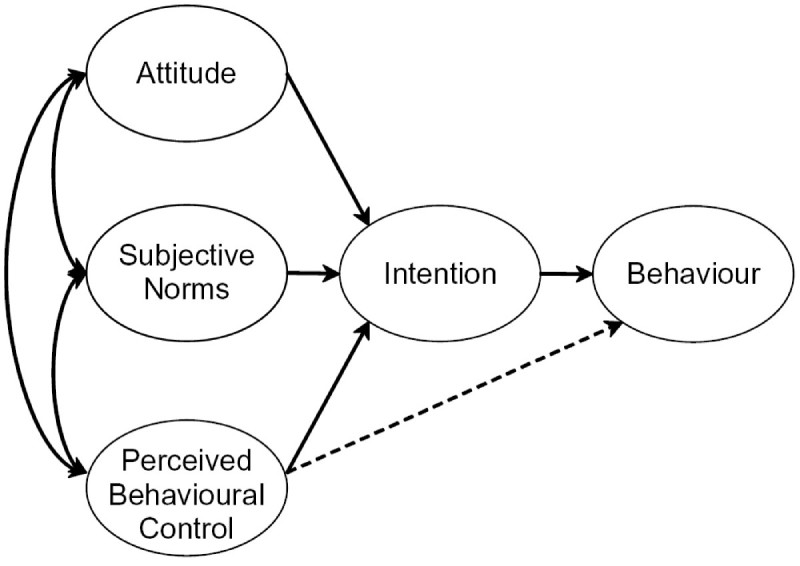

**DPB and the Theory of Planned Behavior**

The theory of planned behavior can be directly applied in the domain of disaster risk reduction. The behavior of interest for present purposes is DPB. According to Ajzen^41^, considering DPB as a category of behaviors, not a single action was studied. The behavioral elements of the public readiness index (PRI) were used for defining and assessing the DPB (Table 1)^42^. The validity and reliability of PRI have been shown in previous studies^43^.

It is hypothesized that intentions to do DPB can be predicted from attitudes, subjective norms, and perceived behavioral control with respect to the behavior; and that actually doing DPB can be predicted from intentions and perceptions of behavioral control. The prediction of DPB, however, depends on the chronological stability of intentions and perceived behavioral control^40^. If these variables change prior to observation of the behavior, they can no longer permit accurate prediction. In addition, precise behavioral prediction also depends on the actual perceived behavioral control. Only if perceptions of control are reasonably accurate will a measure of this variable improve prediction of behavioral success.

**Table 1. BPB index: behavioral elements of PRI:** 

1	Preparation of a home disaster supply kit
2	Preparation of a “go” kit for work or car
3	Creation of a family communication plan
4	Designation of a specific meeting place during an emergency
5	Practicing and performing drills for emergency situations
6	Volunteering to help in emergencies
7	Having successfully completed a first aid training in the past 5 years

## Materials and Methods

**Study population and sampling**

This cross-sectional survey was conducted in August 2015. The study population included inhabitants of Tehran who were 18 years and older. 1250 inhabitants were selected in the study through a random multistage sampling method from 22 districts in Tehran. The sample size for each district was calculated to be proportional to the size of the district populations. First, after numbering the blocks, one of the blocks was chosen randomly in each district. At the second stage, moving in a clockwise direction from that corner, all houses up to the next corner were numbered and one of these, the first unit in the sample was also randomly selected. Trained interviewers started from the first selected unit and filled the questionnaire. Then the next three units were systematically skipped and an individual in the fifth household was interviewed and this continued until the end of the block. If the selected block did not include enough samples, the next block was selected for completing the cluster.

The study was approved by the Tehran University of Medical Sciences Research Ethics Committee. Written consent was received from participants. We did not collect any identifying data.

**Questionnaire**

The questionnaire, which took about 30 min to complete, contained a variety of items dealing with DPB. In addition, measures of sociodemographic characteristics were also obtained. All questions of interest for the present study dealt with the DPB. Three items measured intention to perform DPB. Three items were used to assess attitudes toward DPB. For subjective norms four items were used. Three items were also used to assess perception of behavioral control. Self-reports of DPB were assessed by means of 7 questions (Table 1).

**Analysis**

17 of the 1250 questionnaires were invalid because of missing data and so were excluded from subsequent analyses. The data were grouped according to DPB score. The grouped data were subsequently statistically analyzed using independent t-test to compare means of the variables of TPB among prepared and unprepared people. Structural equation modeling^44^ is used to evaluate the fit between the data and the TPB, taking into account random and systematic measurement error, and to estimate the amount of variance in intentions and behavior explained by the model.

## Results

62.3% of participants were male and the mean age of all participants was 44.14 (SD = 12.53). 71.5% of participants had high school or higher education. 34.5% of participants were currently unemployed (including jobless participants, retired, students and housewives). 54% of participants were owner of their home and most of them (82.5%) living in apartments. 83.5% of the households had less than 4 members. 58.4% of the respondents had not experienced any disaster in the past 20 years. Only 16.3% of participants were not heads of households. 68.1% of responders lived in the high or medium risk districts of Tehran. Most of the participants (65%) reported that they were low income earners. Only 10% of the participants had DPB score of 5 or more which defined as prepared persons (Table 2).

Data analysis showed that monthly income level, previous disaster experience, residential district and occupation are demographic factors that influence DPB significantly. However, disaster preparedness was not affected by gender, educational level, number of household members, home type, home ownership and being the head of household.

**Table 2: DPB scores for the study participants:** 

DPB score	Frequency	Percent	Cumulative Percent
0	531	43.1	43.1
1	246	20	63
2	147	11.9	74.9
3	99	8	83
4	87	7.1	90
5	52	4.1	94.2
6	27	2.2	96.4
7	45	3.6	100

Table 3 shows the means and standard deviations of TPB variables in prepared and unprepared people. Higher means show more favorable dispositions. It can be seen that respondents were positively inclined toward doing DPB. They held highly positive attitudes toward DPB, they somewhat believed that their family, friends and colleagues approved of it, they were moderately confident that they could perform it, and they moderately intended to do DPB. In contrast, self-reported doing DPB was relatively low. Only 10.0% of the respondents reported doing DPB, while 43.1% reported doing so almost never. Clearly, many people who intended to do DPB in actuality failed to do so. Comparison of the means obtained in the prepared and unprepared people shows that overall differences were relatively small.

**Table 3: Means and Standard Deviations of TPB variables in prepared and unprepared people:** Note: N1= 123 (for prepared people); N2= 1110 (for unprepared people)

	Prepared		Unprepared		All Participants	
Latent variable	M	SD	M	SD	M	SD
Attitude toward DPB	5.86	1.54	5.41	1.43	5.46	1.45
Subjective norm	5.29	1.08	4.73	1.08	4.79	1.09
Perceived behavioral control	5.24	1.34	4.82	1.0	4.87	1.05
Intention	4.54	1.18	4.05	0.91	4.10	1.55
Behavior	5.95	0.08	1.07	0.04	1.55	1.93

Independent t- test was used to define any significant difference between prepared and unprepared people. This analysis showed that attitudes of prepared persons toward DPB were significantly more positive than unprepared ones (t= 3.29, p<0.001). It also showed that the prepared persons perceive more social pressure than unprepared ones to perform DPB (t= 5.40, p<0.001). In addition, the people who were prepared had more perceived behavioral control to do DPB when compared to the unprepared people (t= 3.34, p<0.001). Intention and behavior were also significantly different between prepared and unprepared people.

We examined the capacity of the theory of planned behavior to account for intentions to do DPB and its ability to predict actual behavior. Two structural equation models were evaluated: the first relies on the data from the unprepared persons, the second on data collected from the prepared people.

**Unprepared people**

The first structural model to be evaluated examines the associations between attitudes, subjective norms, perceptions of behavioral control, and intentions assessed in the unprepared people, as well as the effects of intentions and perceived behavioral control on reported DPB. This model, as well as the subsequent model, were evaluated using IBM-SPSS AMOS (Version 24.0). This software enables us to specify, estimate, assess and present models to show hypothesized relationships among variables. It lets us build models more accurately than with standard multivariate statistical techniques.Except for DPB, all variables in TPB were assessed by multiple indicators, enabling detection and control for random and non-random measurement error.

The chi-square goodness-of-fit test was not significant, χ2 (70, N =1110) = 17.21, p < 0.20, and the goodness-of-fit index^44^ of 0.98 indicated that there was a very good fit between model and data. Additional goodness-of-fit indices corroborated this conclusion: Comparative Fit Index (CFI)= 1.00, standardized Root Mean Squre Residuals (RMR)= 0.04, and Root Mean Squre Error of Approximation (RMSEA)= 0.00, p = 1.0. Attitudes, subjective norms, and perceived behavioral control accounted for 56.3% of the variance in intentions to do DPB. The measures of these variables were all obtained in the unprepared people. In contrast, only 10.5% of the variance in behavior was accounted for by the model’s two predictors, intentions and perceptions of behavioral control.

Figure 2 shows the path coefficients in the completely standardized solution. The relatively high factor loadings of the indicators imply that the measures had a satisfactory degree of internal consistency. All structural relations were significant at p < 0.05, except for the paths from perceived behavioral control to behavior.

In sum, the results for the unprepared people demonstrated a good fit between the TPB and the obtained data. The theory accounted for a considerable proportion of variance in intentions to do DPB, but the people actually accomplished their intentions depended on perceived behavioral control. The more control they believed they had, the more likely they were to do DPB in accordance with their intentions. Perceived behavioral control did not have a significant effect on behavior for the unprepared participants. As an alternative, its effect was found to depend on intention. Doing DPB increased with perceived control only for respondents who intended to do DPB consistently.

**Prediction of intentions to do DPB and actual doing DPB. Standardized coefficients in the TPB – Unprepared people.**
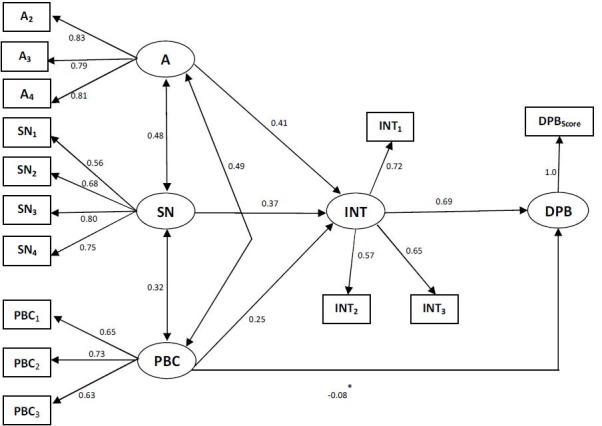
*Coefficient not significant. A= attitude toward behavior; SN= subjective norms; PBC= perceived behavioral control; INT= intention; DPB= disaster preparedness behavior

**Prepared people**

The next structural model examines the associations between attitudes, subjective norms, perceptions of behavioral control, and intentions assessed in the prepared people, as well as the effects of intentions and perceived behavioral control on DPB reported at the same point in time.

As prepared people had more information about DPB, it is expected that their behaviors will have brought expressed attitudes, subjective norms, perceptions of control, and intentions more in line with the actual preceding behavior. The consequence leading to stronger structural relations in the paths leading to DPB. The results of the structural equation analysis support these expectations.

The chi-square goodness-of-fit measure, χ2 (70, N=123) = 35.85, p< 0.20, and the goodness-of-fit index (GFI = 0.96) indicate a very good fit between model and data. Additional goodness-of-fit indices indicated alike results: CFI=0.99, RMR= 0.04**, **and RMSEA=0.02, p < 0.87**. **Attitudes, subjective norms, and perceived behavioral control accounted for 32.0% of the variance in intentions to do DPB. This estimate is unexpectedly lower than in the unprepared people, where 56.4% of the variance in intentions was accounted for, but it is still of suitable magnitude. By way of contrast, for prepared people, the results showed the expected betterment in the prediction of DPB. Whereas only 10.5% of the behavioral variance was explained using data from the unprepared people, with data from the prepared people, 62.8% of the variance in behavior was accounted for. Furthermore, the results indicated perceived behavioral control made a significant contribution to the prediction of DPB, as can be seen in Figure 3.

The factor loadings of the indicators of constructs again showed satisfactory convergence. The ordering of the structural coefficients in the prepared people was the same as in the unprepared ones. Attitude was the most dominating factor in shaping intention to do DPB, followed by subjective norm and perceived behavioral control. The amount of the coefficients, however, was generally smaller than in the unprepared people (Figure 2).

**Prediction of intentions to do DPB and actual doing DPB. Standardized coefficients in the TPB –Prepared people.**
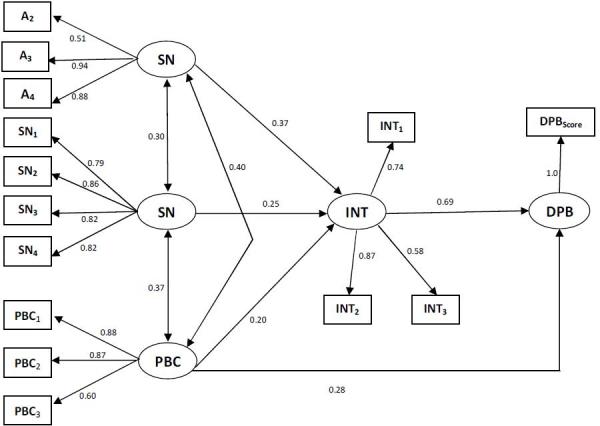
A= attitude toward behavior; SN= subjective norms; PBC= perceived behavioral control; INT= intention; DPB= disaster preparedness behavior

## Discussion

The present study used the TPB to examine doing DPB in a representative sample of Tehran inhabitants and we attempted to predict DPB in them. We relied on the cross-sectional data available at the time of study.

Attitudes and subjective norms and perceptions of behavioral control were found to have significant effects on intentions. The data were examined in both unprepared and prepared people that revealed a more complex picture. There were statistically significant differences between the prepared and unprepared people in the variables of TPB (attitudes toward DPB, subjective norms, perceived behavioral control, and intentions).

Another remarkable finding to emerge in the two group analysis had to do with the role of perceived behavioral control. On average, respondents reported a low level of doing DPB, despite their strong intentions to do so. At least two reasons for this inconsistency can be suggested. First, “doing DPB” is open to interpretation regarding the definition of preparedness. Second, and of greater interest for present purposes, the discrepancy between intentions and behavior may be attributable to unrealistic perceptions of control. Respondents may underestimate or exaggerate the difficulties involved and develop realistic perceptions of behavioral control only with a considerable amount of direct experience.

Perceived behavioral control expressed in the unprepared people was unrelated to DPB (Figure 2), although it did affect DPB in interaction with intentions.

A final finding of significance has implications for the sufficiency assumption of the theory of planned behavior. According to TPB, the effect of new information on later intentions and behavior is mediated by attitudes, subjective norms, and perceptions of behavioral control. Assessing the variables in the TPB model should thus be adequate to predict intentions and behavior. However, the data showed a direct effect of past behavior on later intentions, unmediated by attitudes, subjective norms, and perceived behavioral control. This finding shows violation of the sufficiency assumption. A methodological explanation would attribute the observed insufficiency to unreliability in the measurement of the theory’s constructs. However, analysis of structural equation model corrects at least for low internal consistency amongst the indicators of TPB latent variables.

One last explanation for the direct effect of precedent behavior on intentions should not go unstated. It is possible, of course, that the TPB does not provide a complete description of the processes that underlie the formation of intentions to do DPB. It is needed to repeat the present study with more representative measures of attitudes, subjective norms, and perceptions of control, and to evaluate the direct and indirect effects of precedent behavior on intentions and DPB.

## Conclusion

The results of the present research have essential practical implications for strategies of intervention. First, DPB was found to be influenced both by intentions and perceptions of behavioral control. Therefore, an effective intervention will not only have to encourage people of the desirability of DPB, but also to provide them with the skills and means to do it. The more powerfully they can be made to feel that they have control on DPB, the more likely they are to perform their intentions. That is, heightened perceived control tends to reinforce people’s motivation to do DPB.

## CORRESPONDING AUTHOR

Mehdi Najafi, MD, MPH, PhD

Email: najafirc@gmail.com

University of Social Welfare & Rehabilitation Sciences. Research Center in Emergency & Disaster Health

## COMPETING INTERESTS

The authors have declared that no competing interests exist.

## FUNDING STATEMENT

The authors received no specific funding for this work.

## DATA AVAILABILITY

All relevant data are in the article.

## References

[1] United Nations International Strategy for Disaster Reduction (UNISDR). Terminology on disaster risk reduction. [7/4/2016]; Available from: http://www.unisdr.org/we/inform/terminology.

[2] Lindell MK, Prater CS. Household adoption of seismic hazard adjustments: A comparison of residents in two states. International Journal of Mass Emergencies and Disasters. 2000;18(2):317-38.

[3] Bradley AT. Handbook to Practical Disaster Preparedness for the Family: Arthur Bradley; 2010. Available from: http://www.amazon.com/Handbook-Practical-Disaster-Preparedness-Edition-ebook/dp/B0089NLHD4.

[4] Paton D. Disaster preparedness: a social-cognitive perspective. Disaster Prevention and Management. 2003;12(3):210-6.

[5] Washburn C, Saunders K. Extension Disaster Education Network (EDEN): Preparing Families for Disaster. Journal of Family and Consumer Sciences. 2010;102(2):61-3.

[6] McIvor D, Paton D. Preparing for natural hazards: normative and attitudinal influences. Disaster Prevention and Management. 2007;16(1):79-88.

[7] Armaş I, Avram E. Patterns and trends in the perception of seismic risk. Case study: Bucharest Municipality/Romania. Natural Hazards. 2008;44(1):147-61.

[8] Jackson EL. Response to Earthquake Hazard The West Coast of North America. Environment and Behavior. 1981;13(4):387-416.

[9] Miceli R, Sotgiu I, Settanni M. Disaster preparedness and perception of flood risk: A study in an alpine valley in Italy. Journal of Environmental Psychology. 2008;28(2):164-73.

[10] Lindell MK, Whitney DJ. Correlates of household seismic hazard adjustment adoption. Risk Analysis. 2000;20(1):13-26. 10.1111/0272-4332.00002 10795335

[11] Mulilis JP, Duval TS. Negative threat appeals and earthquake preparedness: A person relative to event (PrE) model of coping with threat. Journal of Applied Social Psychology. 1995;25(15):1319-39.

[12] Mulilis JP, Lippa R. Behavioral change in earthquake preparedness due to negative threat appeals: A test of protection motivation theory. Journal of Applied Social Psychology. 1990;20(8):619-38.

[13] Duval TS, Mulilis JP. A person relative to event (PrE) approach to negative threat appeals and earthquake preparedness: A field study. Journal of Applied Social Psychology. 1999;29(3):495-516.

[14] McClure J, Allen MW, Walkey F. Countering fatalism: Causal information in news reports affects judgments about earthquake damage. Basic and Applied Social Psychology. 2001;23(2):109-21.

[15] McClure J, Walkey F, Allen M. When earthquake damage is seen as preventable: Attributions, locus of control and attitudes to risk. Applied Psychology. 1999;48(2):239-56.

[16] Paton D, Bajek R, Okada N, McIvor D. Predicting community earthquake preparedness: a cross-cultural comparison of Japan and New Zealand. Natural Hazards. 2010;54(3):765-81.

[17] McClure J. Psychology of perception of risk. New Zealand Science Review. 1998;55(1-2):20-4.

[18] Dixey R. Fatalism', accident causation and prevention: issues for health promotion from an exploratory study in a Yoruba town, Nigeria. Health education research. 1999;14(2):197-208. 10.1093/her/14.2.197 10387500

[19] Flynn J, Slovic P, Mertz C, Carlisle C. Public support for earthquake risk mitigation in Portland, Oregon. Risk Analysis. 1999;19(2):205-16.

[20] Ronan KR, Crellin K, Johnston DM, Finnis K, Paton D, Becker J. Promoting child and family resilience to disasters: Effects, interventions, and prevention effectiveness. Children Youth and Environments. 2008;18(1):332-53.

[21] Dooley D, Catalano R, Mishra S, Serxner S. Earthquake Preparedness: Predictors in a Community Survey. Journal of Applied Social Psychology. 1992;22(6):451-70.

[22] Russell LA, Goltz JD, Bourque LB. Preparedness and hazard mitigation actions before and after two earthquakes. Environment and Behavior. 1995;27(6):744-70.

[23] Solberg C, Rossetto T, Joffe H. The social psychology of seismic hazard adjustment: re-evaluating the international literature. Natural Hazards and Earth System Science. 2010;10(8):1663-77.

[24] Paton D, Smith LM, Johnston D. Volcanic hazards: risk perception and preparedness. New Zealand Journal of Psychology. 2000;29(2):86-91.

[25] Paton D. Disaster resilience: integrating individual, community, institutional and environmental perspectives. Disaster resilience: An integrated approach. 2006:306-19.

[26] Paton D, McClure J, Bürgelt PT. Natural hazard resilience: The role of individual and household preparedness. Disaster resilience: An integrated approach. 2006:105-27.

[27] Mileti DS, O'Brien PW. Warnings during disaster: Normalizing communicated risk. Soc Probs. 1992;39:40.

[28] Spittal M, McClure J, Siegert R, Walkey F. Optimistic bias in relation to preparedness for earthquakes. Australasian Journal of Disaster and Trauma Studies. 2005;1:1-10.

[29] Paton D. Preparing for natural hazards: the role of community trust. Disaster Prevention and Management. 2007;16(3):370-9.

[30] Lindell MK, Prater CS. Assessing community impacts of natural disasters. Natural Hazards Review. 2003;4(4):176-85.

[31] Paton D, Millar M, Johnston D. Community resilience to volcanic hazard consequences. Natural Hazards. 2001;24(2):157-69.

[32] Mileti DS, Darlington J. Societal response to revised earthquake probabilities in the San Francisco Bay area. International Journal of Mass Emergencies and Disasters. 1995;13(2):119-45.

[33] Floyd DL, Prentice Dunn S, Rogers RW. A meta-analysis of research on protection motivation theory. Journal of Applied Social Psychology. 2000;30(2):407-29.

[34] Mulilis JP. Social considerations of disaster resistant technology: The person-relative-to-event (PrE) model of coping with threat. The Journal of Urban Technology. 1996;3(3):59-70.

[35] Lindell MK, Perry RW. Household adjustment to earthquake hazard a review of research. Environment and Behavior. 2000;32(4):461-501.

[36] Lindell MK, Perry RW. The protective action decision model: theoretical modifications and additional evidence. Risk Analysis. 2012;32(4):616-32. 10.1111/j.1539-6924.2011.01647.x 21689129

[37] Ajzen I. From intentions to actions: A theory of planned behavior. In: Kuhl J, Bekmann J, editors. Action control from cognition to behavior. Berlin: Springer Verlag; 1985:11-40.

[38] Ajzen I. Theory of planned behavior. In: Van Lange PAM, Kruglanski AW, Higgins ET, editors. Handbook of Theories of Social Psychology: Vol One. London: SAGE Publications; 2012: 438-459.

[39] Ajzen I. The theory of planned behavior. Organizational behavior and human decision processes. 1991;50(2):179-211.

[40] Ajzen I. Perceived Behavioral Control, Self-Efficacy, Locus of Control, and the Theory of Planned Behavior. Journal of Applied Social Psychology. 2002;32:665-83.

[41] Fishbein M, Ajzen I. Predicting and changing behavior: The reasoned action approach: Taylor & Francis; 2011.

[42] Najafi M, Ardalan A, Akbarisari A, Noorbala A A, Jabbari H. Demographic Determinants of Disaster Preparedness Behaviors Amongst Tehran Inhabitants, Iran. PLOS Currents Disasters. 2015. 10.1371/currents.dis.976b0ab9c9d9941cbbae3775a6c5fbe6 PMC4697750 26767148

[43] The council for excellence in government. Are we ready? Introducing the public readiness index. 2006. Available from: https://www.citizencorps.fema.gov/downloads/pdf/ready/pri_report.pdf

[44] Weston R, Gore PA. A brief guide to structural equation modeling. The Counseling Psychologist. 2006;34(5):719-51.

